# Pharmacokinetic profiling of limnetrelvir in non-Japanese and Japanese populations: results of two phase 1 single- and multiple-dose studies

**DOI:** 10.1128/aac.01443-25

**Published:** 2026-03-23

**Authors:** Ekram A. Chowdhury, Christine M. Lee, Janki M. Desai, Izna Ali, Amelia Orejudos, Shelly V. Gupta, Christopher J. Ocampo, Michael G. Miller, Jayanthy Jayanth, Jeffrey M. Schmidt, Ahmed Hamed Salem, Nael M. Mostafa

**Affiliations:** 1 AbbVie Inc. 359181 , North Chicago, Illinois, USA; 2 Department of Clinical Pharmacy, Ain Shams University, Cairo, Egypt; Chinese Academy of Medical Sciences and Peking Union Medical College, Beijing, China

**Keywords:** limnetrelvir, COVID-19, pharmacokinetics, coronavirus

## Abstract

COVID-19’s main protease (Mpro) unique structure and function, which are conserved among coronaviruses, make it an attractive target for viral inhibition. Limnetrelvir is a novel Mpro inhibitor intended for once-daily administration. The pharmacokinetics, safety, and tolerability results of two phase 1 studies of limnetrelvir are reported herein. These studies consisted of a first-in-human, single-, and multiple-ascending dose study in healthy non-Japanese participants and a single- and multiple-dose study in healthy Japanese participants. Across both studies, a total of 104 participants received limnetrelvir single doses ranging from 200 to 800 mg and multiple doses from 200 to 800 mg QD for 10 days. The harmonic mean half-life of limnetrelvir ranged from 6 to 21 h following a single dose. No clinically significant differences in exposures were observed between Japanese and non-Japanese participants. Limnetrelvir was generally well tolerated in both studies in healthy participants with up to 10 days of dosing, with only mild or moderate adverse events observed.

**CLINICAL TRIALS:**

This study is registered with ClinicalTrials.gov as NCT05691699 and NCT06009237.

## INTRODUCTION

The COVID-19 pandemic had a significant impact on global health, resulting in at least 15 million excess deaths as well as disruptions to the global economy, since its onset in 2019 (1). Immunocompromised individuals were and continue to be at high risk. In an observational study, immunocompromised individuals represented 3.9% of the study population, accounted for 22% of COVID-19 hospitalizations and 24% of COVID-19 deaths in 2022 (2). Multiple vaccines were rapidly developed and deployed for the prevention of COVID-19; however, the rapid emergence of new variants and corresponding surges in infections over the course of the pandemic posed significant challenges with updating variant-specific antigens in vaccines (3). Additionally, individuals who were infected post-vaccination produced antibodies against COVID-19 with low neutralizing capacity and breadth against circulating variants, indicating poor immunity (4). Several other treatment options have been considered, including monoclonal antibodies, antiviral drugs, and immunomodulatory drugs. However, the utility of these treatment options is limited due to low efficacy, high price, and/or adverse events (AEs) (5–7). Thus, it is crucial to develop novel treatments that are effective against the currently circulating variants as well as any potential future COVID-19 variants or coronavirus-mediated infections.

COVID-19’s main protease (Mpro/3CL protease) is responsible for cleaving polyproteins (pp1a and pp1ab), resulting in shorter, nonstructural proteins that are crucial for viral replication (8). Mpro’s unique structure and function, which are conserved among coronaviruses, make it an attractive drug development target for antiviral drugs. Inhibiting the activity of Mpro can disrupt viral replication, potentially halting the progression of the infection and ensuring broad efficacy against different coronaviruses. Since there are no human protein homologs (equivalent structures) to the viral Mpro enzyme and mammalian protease enzymes have different substrate specificity, antiviral protease inhibitors targeting Mpro are anticipated to have minimal off-target effects (9).

Nirmatrelvir is an Mpro inhibitor that demonstrated effectiveness against COVID-19 when co-administered with ritonavir (included as a pharmacokinetic [PK] enhancer) (10). It is indicated for the treatment of mild-to-moderate COVID-19 in adults who are at high risk for progression to severe COVID-19, including hospitalization or death. Nirmatrelvir is extensively metabolized by cytochrome P450 (CYP3A) and has a short half-life of approximately 2 h when administered alone, compared to 7 h when administered with ritonavir (11, 12). Due to the inclusion of ritonavir (a strong CYP3A inhibitor) as a PK enhancer, nirmatrelvir has significant drug-drug interaction (DDI) liabilities, which presents serious challenges for patients with comorbidities (13). This represents a significant unmet need and potential opportunity for clinical development of novel Mpro inhibitors with improved PK and DDI liability profiles.

Several Mpro inhibitors are currently under clinical development (9). Limnetrelvir is a novel Mpro inhibitor intended to treat currently circulating COVID-19 variants of concern and to be effective against any potential future pandemic caused by coronaviruses. No adverse effects were identified after a 2-week repeat dose GLP toxicity up to the highest limnetrelvir dose (300 mg/kg in the rat and 300 mg/kg in the dog). Here, we report the results from two phase 1 studies of limnetrelvir conducted in healthy non-Japanese (Study 1) and Japanese participants (Study 2).

## MATERIALS AND METHODS

### Study designs

The studies reported herein were conducted in accordance with the International Council for Harmonization (ICH) guidelines, applicable regulations, and guidelines governing clinical study conduct, as well as ethical principles originating from the Declaration of Helsinki. The study protocols were approved by the Institutional Review Boards/Ethics Committees boards (Advarra, Columbia, MD, USA) of the study sites (see Table S1), and all participants gave written informed consent prior to participation in the studies. Study 1 was conducted between January 03, 2023, and October 23, 2023, at the AbbVie Clinical Pharmacology Research Unit in Grayslake, IL, USA. Study 2 was conducted between August 23, 2023, and October 30, 2023, at Anaheim Clinical Trials in Anaheim, CA, USA.

Both studies consisted of two parts, designated by “A” and “B.” Study 1 was a first-in-human (FIH), randomized, double-blind, placebo-controlled, single-ascending dose (SAD) study (Study 1A) and a multiple ascending dose (MAD) study (Study 1B) of limnetrelvir in healthy participants comprising a non-Japanese population. Study 2 was a phase 1, randomized, double-blind, placebo-controlled single-dose (Study 2A) and multiple-dose (Study 2B) study in Japanese participants. Study designs for Study 1 and Study 2 are provided in Fig. S1.

In Study 1A, single oral doses of 200, 400, and 800 mg of limnetrelvir IR capsules were administered to Groups 1, 2, and 3, respectively. Single doses of 800 and 200 mg of limnetrelvir IR tablets were administered to Groups 4 and 5, respectively. Each dose group consisted of eight participants, where the participants were randomized to receive either limnetrelvir at the specified dose or a matching placebo at a 3:1 ratio. Participants were given a standard moderate-fat meal 30 min prior to dosing for Groups 1 to 3, whereas Groups 4 and 5 were dosed under fasting conditions. Participants were confined at the study site for 5 days and received standardized meals on intensive PK sample collection days.

In Study 1B, multiple oral doses of 200 and 800 mg of limnetrelvir IR capsules were administered to Groups 1 and 2, respectively. Multiple doses of 200, 400, and 600 mg of limnetrelvir IR tablets were administered to Groups 3, 4, and 5, respectively. Each dose group consisted of eight participants, where once-daily (QD) doses were given for 10 days (apart from MAD Group 5, where participants were dosed until day 9). The participants were randomized to receive either limnetrelvir at the specified dose or a matching placebo at a 3:1 ratio. Participants were given a standard moderate-fat meal 30 min prior to dosing in Groups 1 and 2, whereas Groups 3–5 were dosed under fasting conditions. Participants were confined at the study site for 14 days and received standardized meals on intensive PK sample collection days.

The starting dose (200 mg) and dose range (200–800 mg) of limnetrelvir evaluated in Study 1 were based on the no-observed-adverse-effect–levels (NOAEL) determined in preclinical toxicology studies, in accordance with regulatory guidance (14). The decision to escalate to the next dose was based on a review of all available safety and PK data from the preceding group(s). Escalation to doses above 288 mg (the maximum recommended starting dose) was done after all of the following criteria were met: (i) the exposures (area under the plasma concentration time curve [AUC]) in the lower dose group were below 207 μg·hr/mL (the exposure observed in rats at the 300 mg/kg/day dose in the 2-week GLP toxicology study); (ii) the predicted exposures (AUC) of the higher dose were below 207 μg·hr/mL; (iii) the increase in dose was no more than 4-fold higher than the dose from the lower dose group; (iv) if the increase in dose was more than 2-fold higher than the dose from the lower dose group, the predicted exposures (AUC) of the higher dose were no more than approximately 2-fold of the exposures observed in the lower dose group; and (v) there were no safety and tolerability concerns from completed dose groups that would prohibit dose escalation.

In Study 2A, single oral doses of 200 and 400 mg of limnetrelvir IR tablets were administered to Groups 1 and 2, respectively. In Study 2B, multiple 400 mg doses of limnetrelvir IR tablets were administered QD for 10 days to Group 1. Each dose group consisted of eight participants, where the participants were randomized to receive either limnetrelvir at the specified dose or a matching placebo at a 3:1 ratio. Study groups were conducted in parallel. Doses for Study 2 were not expected to exceed exposures previously shown to be safe and tolerated in Study 1 and were selected based on the exposures achieved in Study 1 with consideration of safety and tolerability data.

### Participants

Healthy adult male and female participants were eligible to enroll in Studies 1 and 2 if they were 18–65 years of age inclusive, with a body mass index (BMI) within 18.0–32 kg/m^2^, and were judged to be in good general health. To be eligible to enroll in Study 2, male or female participants had to be between 18 and 65 years of age and first- or second-generation Japanese of full Japanese parentage. First-generation participants must have been born in Japan to two parents, with also four grandparents born in Japan of full Japanese descent. Second-generation participants born outside of Japan must have had two parents and four grandparents born in Japan of full Japanese descent. All participants were required to maintain a typical Japanese lifestyle, including consuming a typical Japanese diet. Additional eligibility criteria are discussed in the Supplemental Methods.

### PK sampling and bioanalytical methods

The intensive PK sampling schedule for Study 1A and Study 2A for limnetrelvir assay was selected to ensure that the absorption, distribution, and terminal phase of limnetrelvir were appropriately captured. Blood samples were collected prior to dosing (0 h) and at 0.5, 1, 1.5, 2, 3, 4, 5, 6, 8, 12, 15, 24, 30, 36, 48, 60, and 72 h after single-dose administration. In Studies 1B and 2B, intensive PK sampling days were performed on days 1 and 10, where blood samples were collected at similar time points as studies 1A and 2A—up to 24 h for day 1 and up to 72 h for day 10. Additional PK samples were also collected to assess trough concentrations on study days in between the intensive PK sampling days (days 1 and 10) for Studies 1B and 2B. Plasma concentrations of limnetrelvir were determined using a validated liquid chromatography and tandem mass spectrometry (LC–MS/MS) bioanalytical method with a lower limit of quantitation (LLOQ) of 5.00 ng/mL. The plasma overall precision (coefficient of variation) was less than or equal to 9.1%, and the overall accuracy (percent bias) was between −2.7% and 10.4% across both parts of each study. Additional information on blood sample collection and analysis can be found in the Supplemental Methods.

### PK and statistical analysis

Noncompartmental analysis of PK parameters for limnetrelvir was performed using Phoenix WinNonlin (Pharsight, A Certara Company, St. Louis, Missouri, USA). The calculated parameters included maximum plasma concentration (*C*_max_); time to *C*_max_ (*T*_max_); plasma concentration at 24 h after dosing (*C*_trough_; for Study 1B and 2B); AUC from time 0 to the time of the last measurable concentration (AUC_last_), from time 0 to infinite time (AUC_inf_) for single doses, and over a 24-hour dosing interval (AUC_tau_) for multiple doses; and the terminal elimination half-life (*t*_1/2_). Accumulation ratios for AUC_tau_ were calculated as the ratios of the respective parameter values on the last dosing day (day 10 for Studies 1B and 2B) to those on day 1. Statistical analyses were performed using SAS (SAS Institute Inc., Cary, North Carolina, USA). Dose proportionality of limnetrelvir was assessed using ANCOVA on the PK parameters. Analyses were performed on the natural logarithms of dose-normalized *C*_max_ and dose-normalized AUC_inf_ for Studies 1A and 2A. To assess the attainment of steady state of limnetrelvir in Studies 1B and 2B, for each dose group, a repeated-measures ANOVA was performed on the pre-dose concentration measurements from days 2–6 to 8–10.

### Safety assessments

Safety was monitored throughout the two studies with clinical and laboratory evaluations, including the collection of AEs, physical examinations, vital signs, 12-lead electrocardiogram, and clinical laboratory testing (hematology, chemistry, and urinalysis). AEs reported by one or more participants in the limnetrelvir or placebo groups in Study 1 and Study 2 are presented in Tables S2 to S4. AEs considered by the investigator to have a reasonable possibility of relationship to the study drug or no reasonable possibility of relationship to the study drug are referred to as “study drug-related” or “not study drug-related,” respectively.

## RESULTS

### Participant demographics and disposition

A summary of the participant demographics is provided in Table 1. In Study 1, 78 of 80 enrolled participants completed the study; two participants prematurely discontinued from the study due to AEs after dosing on day 9 in Study 1B. In Study 2, all 24 enrolled participants in the study completed the study.

**TABLE 1:** Participant demographics for Study 1 and Study 2^*a*^

	Study 1 Mean ± SD	Study 2 Mean ± SD
	**Part A: Single-ascending dose in non-Japanese participants**	**Part A: Single dose in Japanese participants**
N	40	16
Age (years)	48.9 ± 11.2	46.4 ± 8.48
Weight (kg)	81.0 ± 12.3	65.3 ± 10.0
Height (cm)	175 ± 9.6	170 ± 8.8
BMI (kg/m^2^)	26.2 ± 2.8	22.6 ± 2.5
Sex	32 males (80%), 8 females (20%)	14 males (87.5%), 2 females (12.5%)
Race	27 White (67.5%), 7 Black (17.5%), 6 multi-race (15%)	16 Asian (100%)
	**Part B: Multiple-ascending dose in non-Japanese participants**	**Part B: Multiple dose in Japanese participants**
*N*	40	8
Age (years)	44.4 ± 10.2	52.5 ± 5.63
Weight (kg)	82.0 ± 10	71.8 ± 10
Height (cm)	173 ± 8.3	170 ± 10.8
BMI (kg/m^2^)	27.2 ± 2.4	24.9 ± 1.6
Sex	30 males (75%), 10 females (25%)	6 males (75%), 2 females (25%)
Race	23 White (57.5%), 15 Black (37.5%), 1 Asian (2.5%), 1 multi-race (2.5%)	8 Asian (100%)

^*a*^ BMI, body mass index; *N*, number of participants; Sex, biological.

### Single-dose PK in non-Japanese and Japanese participants

**Fig 1:**
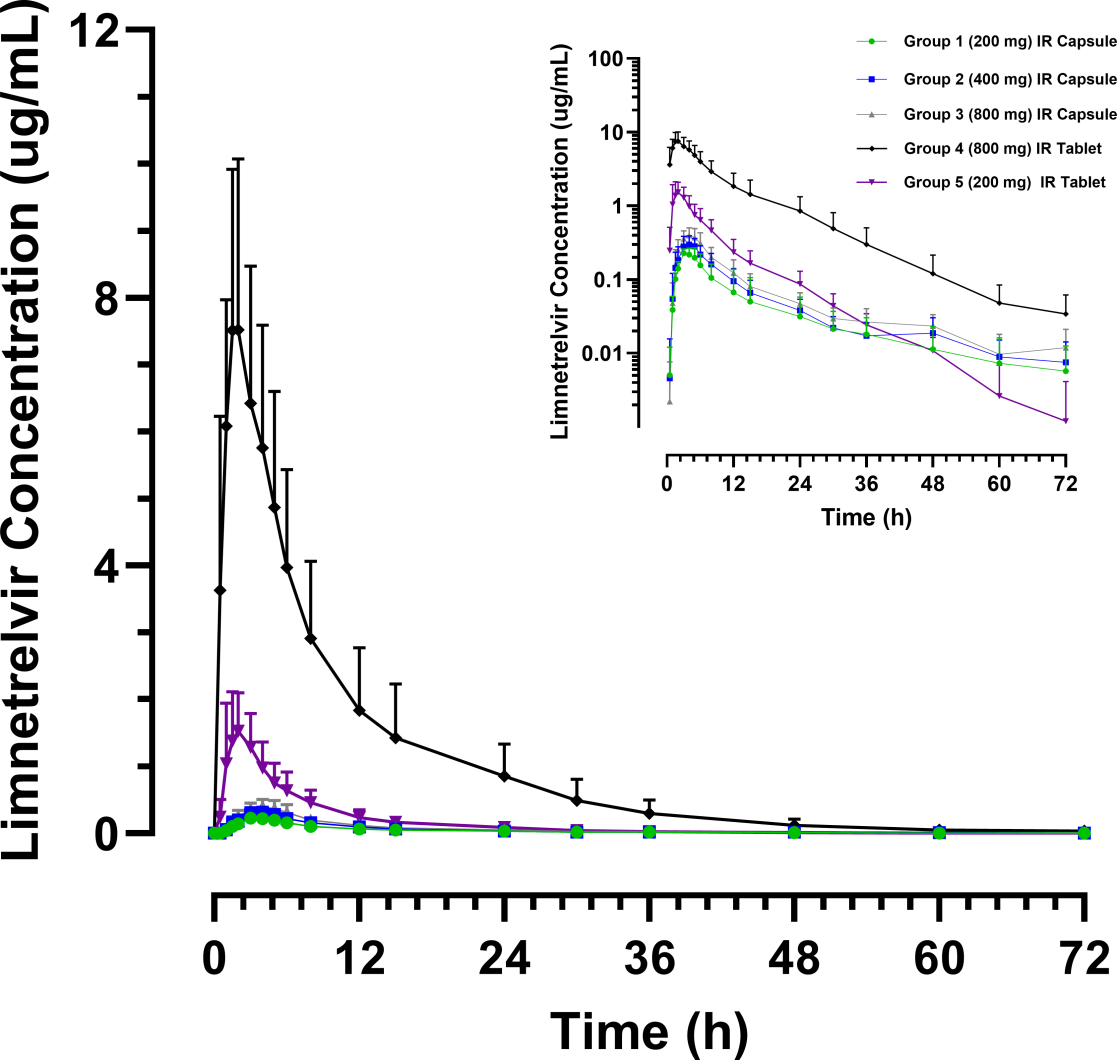
Plasma concentration-time profiles for limnetrelvir administered to the single-ascending dose (SAD) groups in healthy non-Japanese participants from Study 1A. The primary plot is shown on a linear scale, and the inset shows the same data on a semi-log scale. IR, immediate release.

Data of all participants who received a single dose of limnetrelvir (*N* = 40 in Study 1A and *N* = 16 in Study 2A) were included in the PK analyses. Mean plasma concentration–time profiles for limnetrelvir are presented in Fig. 1. Following single oral doses of limnetrelvir IR capsules ranging from 200 to 800 mg, the harmonic mean *t*_1/2_ ranged from ~17 to 21 h, and the median *T*_max_ ranged from 3.0 to 4.5 h. The harmonic mean *t*_1/2_ for single oral doses of limnetrelvir IR tablets dosed from 200 to 800 mg ranged from ~6 to 9 h, and the median *T*_max_ ranged from 1.5 to 2.0 h. Single-dose PK parameters of limnetrelvir from Studies 1A and 2A are presented in Table 2. Limnetrelvir IR capsule exposures (AUC_inf_ and *C*_max_) increased in a less-than-dose-proportional manner (*P* < 0.005) across the 200 mg to 800 mg dose range, whereas the limnetrelvir IR tablet produced a dose-proportional increase in exposures (*P* > 0.05). This is also demonstrated in the dose-normalized exposures of limnetrelvir shown in Fig. 3A through D.

**TABLE 2:** Pharmacokinetic parameters following single-dose administration of limnetrelvir in healthy non-Japanese participants from Study 1A and healthy Japanese participants from Study 2A^*a*^

	Study 1A: FIH SAD groups (non-Japanese population)					Study 2A: Single-dose groups (Japanese population)	
PK parameters (units)	Group 1: 200 mg IR capsule	Group 2: 400 mg IR capsule	Group 3: 800 mg IR capsule	Group 4: 800 mg IR tablet	Group 5: 200 mg IR tablet	Group 1: 200 mg IR tablet	Group 2: 400 mg IR tablet
*N*	6	6	6	6	6	6	6
*C*_max_ (µg/mL)	0.236 (0.24, 20.3)	0.303 (0.313, 26)	0.374 (0.383, 24)	7.27 (7.71, 33)	1.53 (1.66, 42.5)	1.88 (2.14, 50)	5.96 (6.63, 45)
*T*_max_^*b*^ (h)	3.0 (3.0–5.0)	4.5 (1.5–5.0)	4.0 (4.0–5.0)	1.5 (1.5–2.0)	1.75 (1.0–2.0)	2.0 (1.0–2.0)	1.0 (0.5–2.0)
*t*_1/2_^*c*^ (h)	16.9 (9.51)	21.8 (9.2)	21.2 (5.2)	8.82 (1.94)	8.38 (5.42)	7.33 (2.31)	5.98 (1.26)
AUC_last_ (µg·h/mL)	2.37 (2.67, 60.9)	3.44 (3.57, 30.3)	4.37 (4.53, 29.1)	68.7 (75.2, 41.4)	10.08 (10.92, 41.3)	12.9 (15.1, 51)	41.9 (47.2, 48)
AUC_inf_ (µg·h/mL)	2.49 (2.88, 59.8)	3.73 (3.87, 31)	4.81 (5, 30.3)	69.2 (75.7, 41.4)	10.23 (11.07, 40.8)	13.0 (15.2, 51)	42.0 (47.3, 48)

^*a*^ AUC, area under the concentration-time curve from time 0 to the time of the last measurable concentration (AUC_last_), from time 0 to infinite time (AUC_inf_), *C*_max_, maximum concentration; *t*_1/2_, terminal elimination half-life; *T*_max_, time to *C*_max_.

^*b*^ Data reported as geometric mean (mean, %CV), except median (min-max).

^*c*^ Data reported as geometric mean (mean, %CV), except harmonic mean (pseudo SD).

### Multiple-dose PK in non-Japanese and Japanese participants

**Fig 2:**
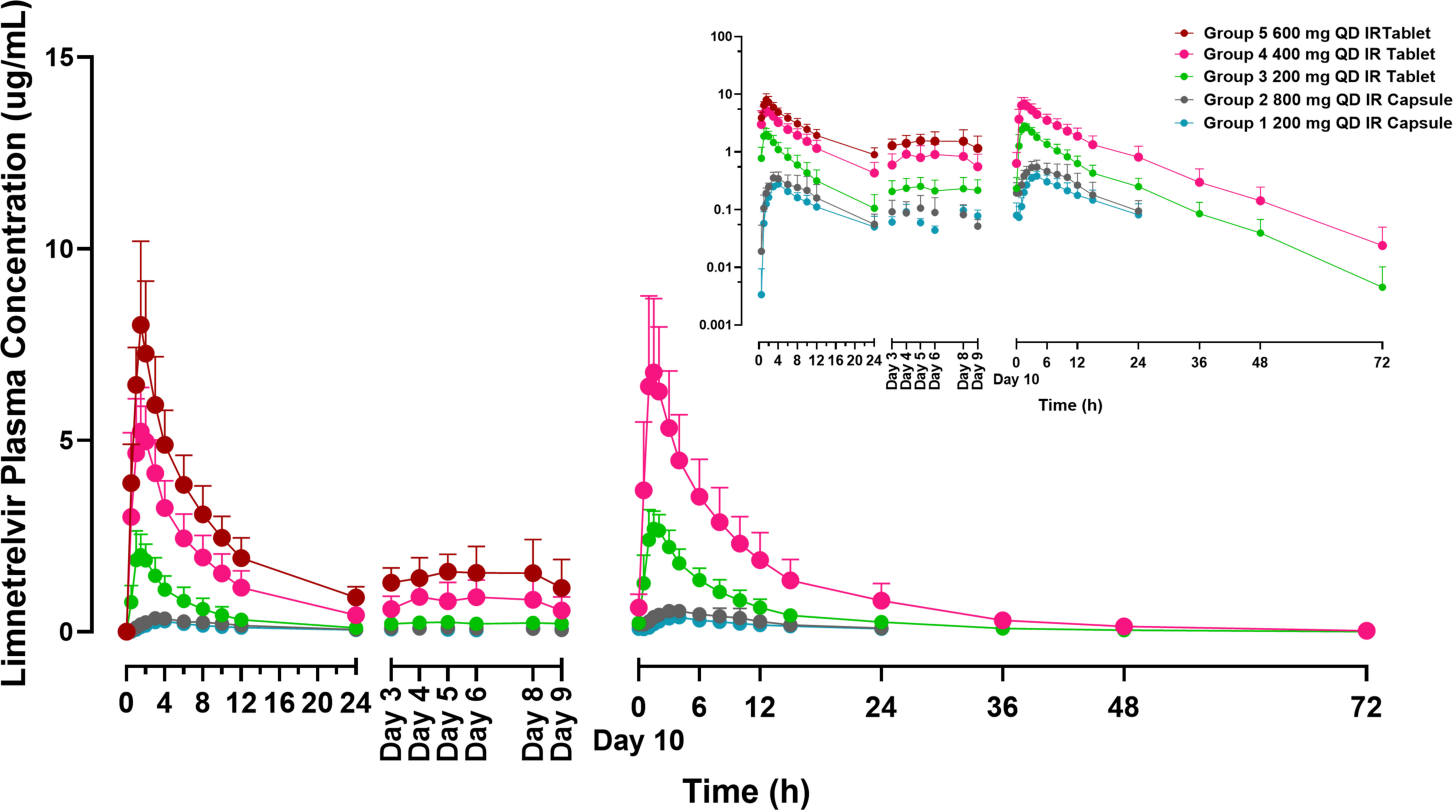
Limnetrelvir plasma concentration-time profiles for multiple-ascending dose (MAD) groups in healthy non-Japanese participants from Study 1B. The primary plot is shown on a linear scale, and the inset shows the same data on a semi-log scale. IR, immediate release; QD, once daily.

Data of all healthy participants who received limnetrelvir were included in the PK analyses (*N* = 40 for Study 1B and *N* = 8 for Study 2B), except for the Study 1 MAD Group 5, day 10 data. The mean plasma concentration–time profiles for limnetrelvir after multiple doses for 10 days QD are presented in Fig. 2. The summarized multiple-dose limnetrelvir PK parameters in healthy participants (day 1 and day 10) from Study 1B and Study 2B are presented in Table 3, respectively. In healthy participants, after multiple doses with IR capsules, limnetrelvir reached peak levels at approximately 3.0 to 4.0 h (median *T*_max_), and the harmonic mean *t*_1/2_ ranged from 13 to 15 h (on day 10). Multiple doses with IR tablets produced limnetrelvir peak plasma concentrations at approximately 1.5–2.0 h (median *T*_max_), and the harmonic mean *t*_1/2_ ranged from 6 to 8 h (on day 10). Repeated-measures analysis of the natural logarithm of *C*_trough_ indicated that steady-state concentrations in healthy participants were attained by day 3 for both IR capsule and tablet formulations. Following administration of limnetrelvir 200–800 mg IR capsules and 200–400 mg IR tablets for 10 days QD, the median accumulation ratio across the different groups ranged between ~1.4-1.9 for both studies.

**TABLE 3:** Pharmacokinetic parameters on day 1 and day 10 following multiple-dose (QD) administration of limnetrelvir for 10 days in healthy non-Japanese participants in Study 1B and Japanese participants in Study 2B^*a*^

	Study 1B: FIH MAD groups (non-Japanese population)					Study 2B: Multiple-dose group (Japanese population)
	Group 1: 200 mg QD IR capsule	Group 2: 800 mg QD IR capsule	Group 3: 200 mg QD IR tablet	Group 4: 400 mg QD IR tablet	Group 5: 600 mg QD IR tablet	Group 1: 400 mg QD IR tablet
PK parameters (units) day 1						
*N*	6	6	6	6	6	6
*C*_max_ (µg/mL)	0.280 (0.283, 15.5)	0.365 (0.375, 25.3)	2.01 (2.08, 30.1)	5.21 (5.31, 21.7)	7.76 (8.01, 27.3)	5.34 (6.01, 58)
*T*_max_^*b*^ (h)	4.0 (3.0–4.0)	3.0 (1.5–4.0)	1.5 (1.0–2.0)	1.5 (1.0–2.0)	1.5 (1.5–1.5)	2.0 (1.0–2.0)
AUC_tau_ (µg·h/mL)	2.91 (2.96, 21.6)	3.72 (4.11, 49.2)	12.5 (13.3, 40.6)	40.0 (41.6, 29.9)	64.1 (65.4, 22.2)	38.1 (43.1, 60)
*C*_trough_ (µg/mL)	0.046 (0.050, 52.1)	0.050 (0.056, 50.1)	0.084 (0.105, 73.2)	0.38 (0.43, 52.1)	0.86 (0.90, 31.2)	0.30 (0.43, 96)
PK parameters (units) day 10						
*C*_max_ (µg/mL)	0.389 (0.394, 16.9)	0.546 (0.569, 31)	2.76 (2.8, 16.7)	6.48 (6.80, 28.9)	NA	8.70 (9.04, 29)
*T*_max_^*b*^ (h)	4.0 (2.0–4.0)	4.0 (3.0–10.0)	1.5 (1.0–2.0)	1.5 (1.0–1.5)		1.0 (1.0–2.0)
*t*_1/2_^*c*^ (h)	12.5 (7.13)	14.8 (9.80)	8.35 (1.90)	7.74 (2.99)		6.13 (1.02)
AUC_tau_ (µg·h/mL)	4.43 (4.57, 26.7)	6.18 (6.77, 45.8)	21.1 (21.8, 26)	56.0 (59.1, 32.0)		73.7 (77.1, 35)
*C*_trough_ (µg/mL)	0.073 (0.081, 56.6)	0.0843 (0.0941, 50.9)	0.23 (0.25, 38.8)	0.695 (0.811, 55.0)		0.796 (0.910, 63)
Rac_(AUCtau)_^*b*^	1.54 (1.35–1.85)	1.74 (1.11–2.13)	1.69 (1.20–2.43)	1.52 (0.789–1.80)		1.90 (1.40–3.10)
Rac_(Cmax)_^*b*^	1.41 (1.05–1.81)	1.51 (1.33–1.69)	1.33 (1.09–1.79)	1.34 (0.703–1.75)		1.60 (1.10–2.40)

^*a*^ AE, adverse event; *C*_max_, maximum concentration; CV, coefficient of variation; FIH, first-in-human; MAD, multiple-ascending dose; Rac, accumulation ratio; *N*, number of participants; NA, data not available since participants released early from confinement due to observed AE; QD, once daily; SD, standard deviation; *T*_max_, time to *C*_max_.

^*b*^ Data reported as geometric mean (mean, %CV), except median (min-max).

^*c*^ Data reported as geometric mean (mean, %CV), except harmonic mean (pseudo SD).

Among the three MAD dose levels assessed using the limnetrelvir IR tablets, only 200 mg QD and 400 mg QD had complete data sets for the extensive PK collection days on day 1 and day 10. The geomean of the AUC_tau_ was found to be 56 µg⋅h/mL at steady state after limnetrelvir IR tablet doses of 400 mg QD, providing safety margins of ~3× and ~4× relative to the NOAEL exposures observed in dogs and rats, respectively. The timing of the occurrence of the AEs associated with chest pain (MAD Group 5, day 9), along with hyperkalemia, led investigators to withhold dosing of limnetrelvir on day 10 and release participants from confinement. Hence, the extensive PK data for MAD Group 5 on day 10 had not been reported.

### Comparison of exposures between non-Japanese and Japanese populations

**Fig 3:**
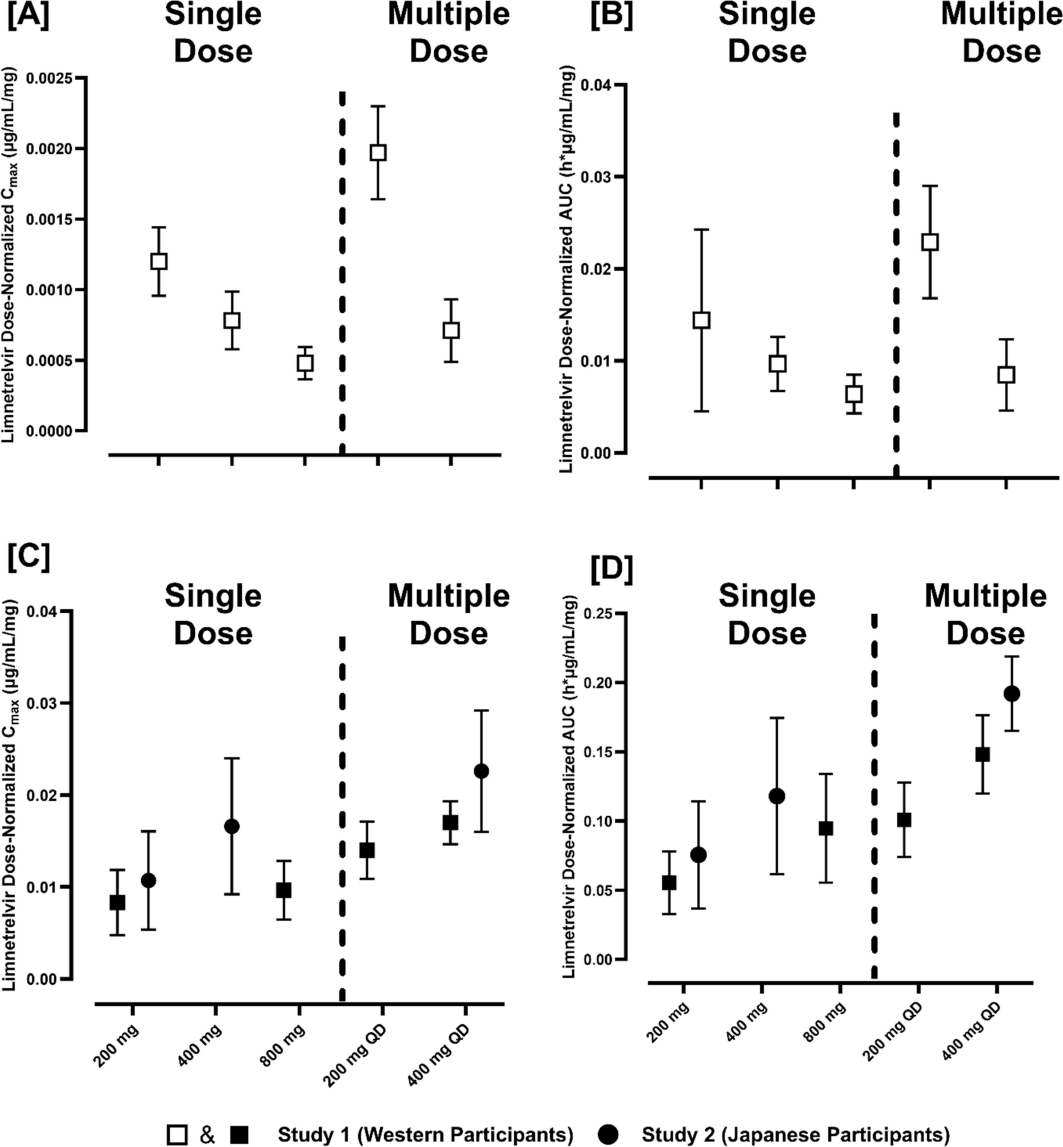
Dose-normalized exposures (*C*_max_ and AUC) of limnetrelvir IR capsules (**A and B**) and IR tablets (**C and D**) after a single dose or multiple doses (10 days QD) in healthy non-Japanese (white and black boxes) or Japanese participants (black circle). AUC, area under the plasma concentration-time curve; *C*_max_, maximum concentration; IR, immediate release; QD, once daily. Data presented as geometric mean ± SD.

A comparison between the exposures of the non-Japanese population (Caucasian, Black, Asian, and mixed-race participants) from the FIH study and the Japanese population from the Japanese PK study at the same dose levels shows ~28–37% higher exposure in Japanese population (*C*_max_ and AUC_inf_) after single or multiple doses. Dose-normalized exposures for the non-Japanese population and Japanese population are shown in Fig. 3.

### Safety

All AEs reported in this manuscript were treatment-emergent (i.e., any event that began or worsened in severity after initiation of study drug through 30 days after the last dose administered). In Studies 1 and 2, single dose administration of limnetrelvir ranging from 200 to 800 mg and multiple-ascending doses of limnetrelvir ranging from 200 mg QD to 600 mg QD (IR tablets) or 800 mg QD (IR capsules) for 10 days was generally well tolerated with all AEs being Grade 1 in severity. A total of seven AEs were reported in Studies 1 and 2. Studies 1A and 1B had 2 and 5 AEs, respectively, while Study 2 had no AEs reported. Two participants were discontinued from study treatment in the limnetrelvir 600 mg QD group due to AEs in Study 1B (MAD Group 5). Limnetrelvir dosing was stopped on day 9 due to hyperkalemia reported with concurrent nonserious AEs of lower extremity weakness, fatigue, and chest pain in one participant. The participant had non-cardiac chest pain, and another participant had a rash later confirmed to be from an arthropod bite. Upon investigation, both AEs were deemed not study-drug related. All laboratory abnormalities were assessed by the investigator or the AbbVie medical monitor as not to be clinically significant, except for hyperkalemia reported in Study 1B for one participant who received limnetrelvir. A medical safety evaluation did not support a causal association between hyperkalemia and limnetrelvir. No clinically significant vital signs or ECG measurements were observed during the study, and no new safety signals were identified in this study.

## DISCUSSION

This is the first report of clinical PK and safety of limnetrelvir, an orally administered small molecule MPro inhibitor for coronavirus. Limnetrelvir was generally well tolerated after single doses up to 800 mg and after multiple doses up to 600 mg QD (IR tablets) and 800 mg QD (IR capsules) for 10 days in healthy participants.

Limnetrelvir is a BCS Class II drug (low solubility and high permeability). Based on *in vitro* assessments, the IR capsules were predicted to have poor solubility. Hence, participants receiving the limnetrelvir IR capsules in the Study 1 SAD and MAD groups were administered limnetrelvir following a moderate-fat meal to overcome the potential solubility limitation. Following a 4-fold dose increase from 200 to 800 mg (Study 1A), limnetrelvir exposures (*C*_max_ and AUC_inf_) increased less than 2-fold. Based on the observed less-than-dose-proportional increases in the SAD groups’ exposures, additional language was incorporated into the dose escalation and stopping criteria section of the protocol. Doses could be escalated in subsequent dose groups by up to 4-fold as long as the predicted exposures for the new dose group increased ≤2-fold compared with the previous dose group and were less than the NOAEL exposures. In the two MAD groups dosed with IR capsules, *C*_max_ and AUC_tau_ at steady state increased by only ~1.4-fold over a 4-fold dose increase from 200 mg to 800 mg QD. The steady-state exposures of limnetrelvir (geomean of AUC_tau_: 6.18 µg·h/mL) following the 800 mg QD dose regimen were 23- and 30-fold lower than the NOAEL exposures observed in GLP toxicology study in dogs (159 µg·h/mL) and rats (207 µg·h/mL), respectively, at a dose of 300 mg/kg/day.

In the interest of expedited clinical development, additional tablet formulation work was completed to overcome the solubility-related limitations of the first capsule formulation. This tablet formulation was then dosed under fasting conditions. Following the administration of a single 200 mg dose of limnetrelvir tablet under fasted conditions, geomean exposures of 2.42 µg/mL and 19.5 µg.h/mL were observed for *C*_max_ and AUC_inf_, respectively. In contrast to the IR capsules, roughly dose-proportional increases in exposure were observed at a 2-fold higher dose of 400 mg limnetrelvir IR tablets (geomean of *C*_max_ of 4.76 µg/mL and geomean of AUC_inf_ of 46.7 µg⋅h/mL).

Assuming linear pharmacokinetics, the predicted exposures at 800 mg were 2-fold below the NOAEL exposures. Thus, the limnetrelvir IR tablet evaluation in the SAD group (Study 1A) was initiated at the 800 mg dose. Group 4 and the 200 mg QD dose were considered the starting dose for the MAD part (Study 1B), Group 3 of the FIH study.

Safety and PK of limnetrelvir were also assessed in a healthy Japanese population (Study 2). Cross-study comparison of single- and multiple-dose groups at the same dose levels demonstrated that exposures (*C*_max_ and AUC_inf_) were slightly higher (~28–37%) in the Japanese population. The small difference in exposures between the two groups, the lower median BMI in Japanese participants compared to the FIH study population, and the observed variability in the data (%CV ranging between 17% and 51% for the exposure metrics) suggest that this is not a clinically significant observation. Thus, no dose adjustments for Japanese participants are warranted.

Interestingly, the geometric mean dose-normalized AUC_tau_ and *C*_max_ of nirmatrelvir were approximately 30% and 21–26% lower, respectively, in Japanese participants compared to exposures observed for non-Japanese participants (15). This is the opposite of what was observed in these limnetrelvir studies. Similarly, the exposure differences for nirmatrelvir were not expected to be clinically significant, and no dose adjustments were recommended (15).

The safety and tolerability profiles were comparable between participants who received limnetrelvir and placebo. In both studies, there were no discontinuations or serious AEs due to limnetrelvir. Limnetrelvir dosing was stopped in the MAD Group 5 on day 9 due to hyperkalemia and concurrent nonserious AEs, which included chest pain in one participant. Further medical evaluation suggested that the participant had non-cardiac chest pain, and no further causal association between hyperkalemia and limnetrelvir was identified.

### Conclusion

Limnetrelvir IR tablets were safe and well tolerated at single doses up to 800 mg and multiple doses up to 600 mg QD in healthy participants. Limnetrelvir IR tablets produced significantly higher and dose-proportional exposures compared to limnetrelvir IR capsules. The PK profile for limnetrelvir IR tablet is suitable for once-daily dosing without coadministration of ritonavir as a PK enhancer. Limnetrelvir exposures (*C*_max_ and AUC_inf_) were slightly higher (~28–37%) in the Japanese population compared to the non-Japanese population. The overall safety and tolerability data from these studies support further clinical development of limnetrelvir in patients with COVID-19 in a phase 2 study.

## Data Availability

AbbVie Inc. is committed to responsible data sharing regarding the clinical trials we sponsor. This includes access to anonymized, individual, and trial-level data (analysis data sets), as well as other information (e.g., protocols, clinical study reports, or analysis plans), as long as the trials are not part of an ongoing or planned regulatory submission. This includes requests for clinical trial data for unlicensed products and indications. These clinical trial data can be requested by any qualified researchers who engage in rigorous, independent, scientific research and will be provided following review and approval of a research proposal, Statistical Analysis Plan (SAP), and execution of a Data Sharing Agreement (DSA). Data requests can be submitted at any time after approval in the US and Europe and after acceptance of this manuscript for publication. The data will be accessible for 12 months, with possible extensions considered. For more information on the process or to submit a request, visit the following link: https://vivli.org/ourmember/abbvie/ then select “Home”.

## SUPPLEMENTAL MATERIAL

Supplemental material aac.01443-25-s0001.docx Tables S1 to S4; Fig. S1.

ASM does not own the copyrights to Supplemental Material that may be linked to, or accessed through, an article. The authors have granted ASM a non-exclusive, world-wide license to publish the Supplemental Material files. Please contact the corresponding author directly for reuse.

## References

[1] Paglino E, Lundberg DJ, Wrigley-Field E, Zhou Z, Wasserman JA, Raquib R, Chen Y-H, Hempstead K, Preston SH, Elo IT, Glymour MM, Stokes AC. 2024. Excess natural-cause mortality in US counties and its association with reported COVID-19 deaths. Proc Natl Acad Sci USA 121:e2313661121. doi:10.1073/pnas.2313661121 38300867 PMC10861891

[2] Evans RA, Dube S, Lu Y, Yates M, Arnetorp S, Barnes E, Bell S, Carty L, Evans K, Graham S, Justo N, Moss P, Venkatesan S, Yokota R, Ferreira C, McNulty R, Taylor S, Quint JK. 2023. Impact of COVID-19 on immunocompromised populations during the Omicron era: insights from the observational population-based INFORM study. Lancet Reg Health Eur 35:100747. doi:10.1016/j.lanepe.2023.100747 38115964 PMC10730312

[3] Flores-Vega VR, Monroy-Molina JV, Jiménez-Hernández LE, Torres AG, Santos-Preciado JI, Rosales-Reyes R. 2022. SARS-CoV-2: evolution and emergence of new viral variants. Viruses 14:653. doi:10.3390/v14040653 35458383 PMC9025907

[4] Suntronwong N, Kanokudom S, Duangchinda T, Chantima W, Pakchotanon P, Klinfueng S, Puenpa J, Thatsanathorn T, Wanlapakorn N, Poovorawan Y. 2025. Neutralization of omicron subvariants and antigenic cartography following multiple COVID 19 vaccinations and repeated omicron non JN.1 or JN.1 infections. Sci Rep 15:1454. doi:10.1038/s41598-024-84138-0 39789099 PMC11718010

[5] Weinreich DM, Sivapalasingam S, Norton T, Ali S, Gao H, Bhore R, Musser BJ, Soo Y, Rofail D, Im J, et al.. 2021. REGN-COV2, a neutralizing antibody cocktail, in outpatients with Covid-19. N Engl J Med 384:238–251. doi:10.1056/NEJMoa2035002 33332778 PMC7781102

[6] Parums DV. 2022. Editorial: Current status of oral antiviral drug treatments for SARS-CoV-2 infection in non-hospitalized patients. Med Sci Monit 28:e935952. doi:10.12659/MSM.935952 34972812 PMC8729033

[7] Drożdżal S, Rosik J, Lechowicz K, Machaj F, Szostak B, Przybyciński J, Lorzadeh S, Kotfis K, Ghavami S, Łos MJ. 2021. An update on drugs with therapeutic potential for SARS-CoV-2 (COVID-19) treatment. Drug Resist Updat 59:100794. doi:10.1016/j.drup.2021.100794 34991982 PMC8654464

[8] Hoang H-D, Naeli P, Alain T, Jafarnejad SM. 2023. Mechanisms of impairment of interferon production by SARS-CoV-2. Biochem Soc Trans 51:1047–1056. doi:10.1042/BST20221037 37199495 PMC10317165

[9] Zagórska A, Czopek A, Fryc M, Jończyk J. 2024. Inhibitors of SARS-CoV-2 main protease (Mpro) as anti-coronavirus agents. Biomolecules 14:797. doi:10.3390/biom14070797 39062511 PMC11275247

[10] Hammond J, Leister-Tebbe H, Gardner A, Abreu P, Bao W, Wisemandle W, Baniecki M, Hendrick VM, Damle B, Simón-Campos A, Pypstra R, Rusnak JM, EPIC-HR Investigators. 2022. Oral nirmatrelvir for high-risk, nonhospitalized adults with Covid-19. N Engl J Med 386:1397–1408. doi:10.1056/NEJMoa2118542 35172054 PMC8908851

[11] Singh RSP, Toussi SS, Hackman F, Chan PL, Rao R, Allen R, Van Eyck L, Pawlak S, Kadar EP, Clark F, Shi H, Anderson AS, Binks M, Menon S, Nucci G, Bergman A. 2022. Innovative randomized phase I study and dosing regimen selection to accelerate and inform pivotal COVID-19 trial of nirmatrelvir. Clin Pharmacol Ther 112:101–111. doi:10.1002/cpt.2603 35388471 PMC9087011

[12] Gerhart J, Cox DS, Singh RSP, Chan PLS, Rao R, Allen R, Shi H, Masters JC, Damle B. 2024. A comprehensive review of the clinical pharmacokinetics, pharmacodynamics, and drug interactions of nirmatrelvir/ritonavir. Clin Pharmacokinet 63:27–42. doi:10.1007/s40262-023-01339-y 38177893 PMC10786959

[13] Igho-Osagie E, Puenpatom A, Williams MG, Song Y, Yi D, Wang J, Berman R, Gu M, He C. 2023. Prevalence of potential drug-drug interactions with ritonavir-containing COVID-19 therapy. J Manag Care Spec Pharm 29:509–518. doi:10.18553/jmcp.2023.22366 36989455 PMC10394216

[14] FDA. 2005. Estimating the maximum safe starting dose in initial clinical trials for therapeutics in adult healthy volunteer. Available from: https://www.fda.gov/media/72309/download

[15] CDER. 2023. Integrated review of PAXLOVID. Available from: https://www.accessdata.fda.gov/drugsatfda_docs/nda/2023/217188Orig1s000IntegratedR.pdf

