# Supplementary material for: Pharmacokinetic profiling of limnetrelvir in non-Japanese and Japanese populations: results of two phase 1 single- and multiple-dose studies
Source: Antimicrob Agents Chemother. 2026 Mar 23;70(5):e01443-25. doi: 10.1128/aac.01443-25 (PMC13148031; doi:10.1128/aac.01443-25)
Supplement: Supplemental material — Tables S1 to S4; Fig. S1. [file aac.01443-25-s0001.docx]

**SUPPLEMENTAL MATERIALS**

**Pharmacokinetic Profiling of Limnetrelvir in Western and Asian Populations: Results of Phase 1 Single and Multiple Dose Studies**

Ekram Ahmed Chowdhury^1*^ PhD, Christine M. Lee^1*^ PhD, Janki M. Desai^1^ PhD, Izna Ali^1#^ PhD, Amelia Orejudos^1^ MSc, Shelly V. Gupta^1^ MD, Christopher J. Ocampo^1^ MD, Michael G. Miller^1^ PharmD, Jayanthy Jayanth MS^1^, Jeffrey M. Schmidt BS^1^, Ahmed Hamed Salem^1^ PhD, FCP, Nael M. Mostafa^1^ PhD

^1^ AbbVie Inc., North Chicago, IL US

*Both authors contributed equally to the manuscript

# affiliation at the time of contribution to this study

**Corresponding author:**

Ekram Ahmed Chowdhury; AbbVie Inc.; Clinical Pharmacology and Pharmacometrics; 1 North Waukegan Road, Dept. R4PK, Bldg. AP31-3; North Chicago, IL; OFFICE: +18479356588; EMAIL: [ekram.chowdhury@abbvie.com](mailto:ekram.chowdhury@abbvie.com); clinpharm.publications@abbvie.com

**Participant Eligibility Criteria**

In addition to the key inclusion criteria listed in the main body of the manuscript, the following eligibility criteria were also required in both studies. Females were required to be of non-childbearing potential, either postmenopausal or surgically sterile, since the impact of Limnetrelvir on pregnancy and reproduction was unknown at the time of the study. Surgically sterile female participants were required to produce a negative urine pregnancy test at screening and a negative serum pregnancy test on Day -1 (day prior to dosing). Sexually active males, with or without a vasectomy, were required to utilize appropriate contraception during participation of the study and for ~93 days after the last dose of study drug. In addition, participants were required to have negative test results for a hepatitis test panel, human immunodeficiency virus (HIV), drugs of abuse, alcohol, and cotinine at the screening visit and on Day -1. Concomitant medications consisting of any investigational drug or live vaccine within 30 days prior to Day 1 (first dose of study drug); non-live vaccine (e.g., mRNA, non-replicating viral vector, protein subunit, etc.) within 2 weeks of Day 1; over the counter and/or prescription medication, vitamins and/or herbal supplements on a regular basis and within 2 weeks prior to Day 1, with the exception of hormonal replacement therapies for females; known inhibitors or inducers of cytochrome P450 (CYP) enzymes within 30 days of Day 1 were prohibited. All prior and concomitant therapies after enrollment and through the last study visit were recorded.

**Pharmacokinetic Samples and Bioanalytical Methods**

Blood samples were collected in evacuated potassium-ethylenediaminetetraacetic acid (K2-EDTA) containing collection tubes. Immediately after collection, the blood samples were inverted approximately 8-10 times to ensure good mixing of the blood and anticoagulant and were placed in an ice bath or chilled cryoblock until centrifugation. Centrifugation was performed between approximately 1100 – 1600 x g for approximately 10 minutes in a refrigerated centrifuge (2°C – 8°C) to separate the plasma. Plasma was aliquoted into clean polypropylene tubes and placed in a freezer maintained at -20°C or colder within 1 hour after centrifugation and stored until they were shipped to Abbvie Inc. (North Chicago, IL, USA) for analysis.

Limnetrelvir was extracted from human plasma samples using liquid-liquid extraction within 2.0 mL 96-well polypropylene plates. The plasma samples were combined with a solution containing the stable labeled isotope, D8-limnetrelvir, and mixed. Limnetrelvir and D8-limnetrelvir were isolated using a mixture of ethyl acetate and hexanes, mixed and centrifuged. A portion of the supernatant was transferred to a clean 1.2 mL 96-well polypropylene plate, evaporated to dryness and reconstituted with a mixture of acetonitrile and water. Chromatographic separation was performed using reversed phase chromatography on a Waters XBridge C8 (2.1 x 30 mm, 5 µm) analytical column with a mobile phase comprised of acetonitrile, methanol, water and formic acid. Detection was performed on a Sciex API5500 triple quadrapole mass spectrometer in positive ion multiple-reaction monitoring (MRM) mode using mass to charge (m/z) transitions of 558 🡪 265 for limnetrlvir and 566 🡪 265 for D8-limnetrelvir. Concentrations of limnetrelvir were quantitated using weighted (1/x^2^) linear least-squares regression generated from human plasma calibration samples with nominal concentrations ranging from 5.00 to 5000 ng/mL.

**Table S1. Study Sites**

| **Study** | **Site Name/Post office address** | **Name and address of IRB** |
| --- | --- | --- |
| **Study 1** | **AbbVie Clinical Pharmacology Research Unit**  480 S. US Highway 45  Grayslake, IL 30030 | **Advarra IRB**  6100 Merriweather Dr.  Suite 600  Columbia, MD 21046 |
| **Study 2** | **Anaheim Clinical Trials**  2441 W. La Palma Ave Suite 140  Anaheim, CA 92801 |  |

**Study Design Schematic**

**Figure S1:** Study design schematic for Study 1 (First-in-human Single and Multiple ascending dose study in healthy western participants) and Study 2 (Single and Multiple dose study in Japanese participants).

**
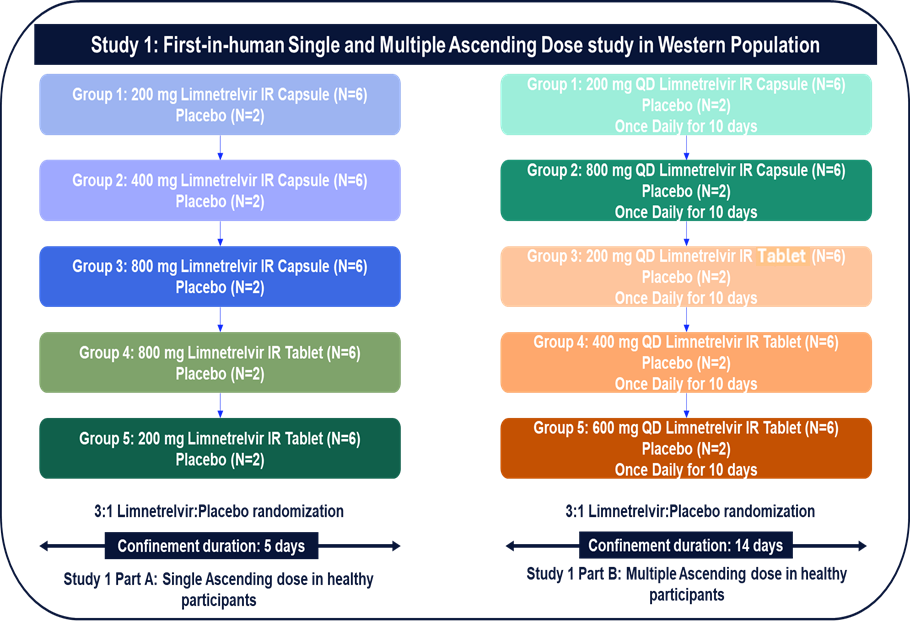
**

**
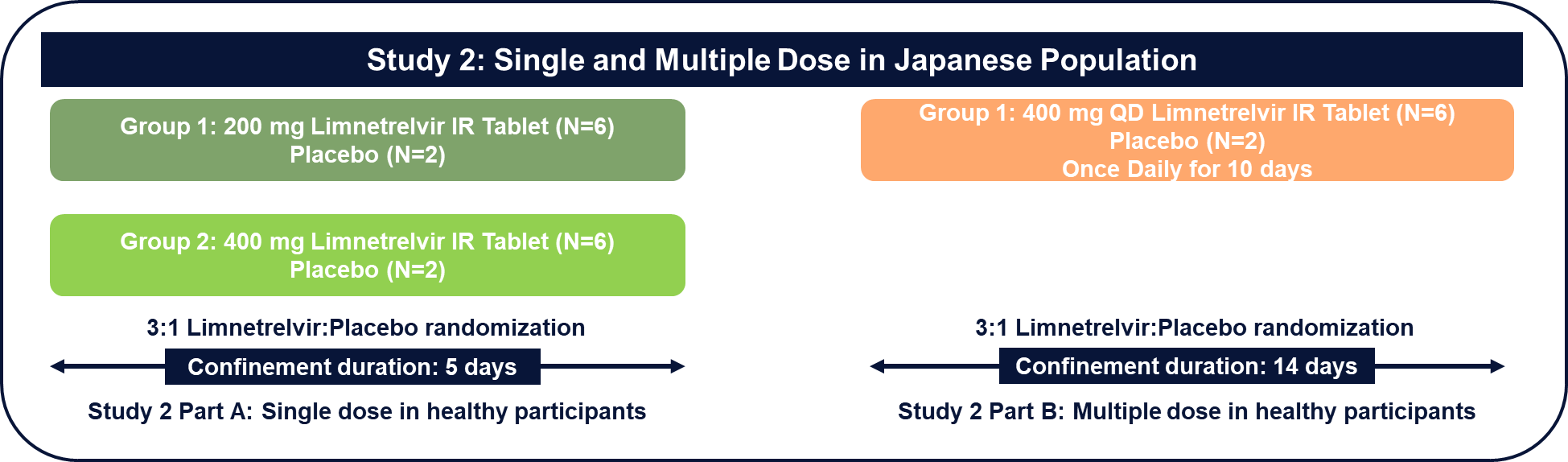
**

**Table S2. Treatment-Emergent Adverse Events Reported by one or more Participants Administered Limnetrelvir or Placebo in Study 1 Part A**

|  | Placebo  (N=10)  N (%) | Group 1  (N=6)  N (%) | Group 2  (N=6)  N (%) | Group 3  (N=6)  N (%) | Group 4  (N=6)  N (%) | Group 5  (N=6)  N (%) | Total  (N=30)  N (%) | Overall  (N=40)  N (%) |
| --- | --- | --- | --- | --- | --- | --- | --- | --- |
| Participants with treatment emergent adverse event (AE) | 2 (20.0) | 2(33.3) | 2(33.3) | 0 | 0 | 0 | 4(13.3) | 6(15.0) |
| AE with reasonable possibility of being related to study treatment | 1(10.0) | 0 | 2(33.3) | 0 | 0 | 0 | 2(6.7) | 3(7.5) |
| Severe AE | 0 | 0 | 0 | 0 | 0 | 0 | 0 | 0 |
| Serious AE | 0 | 0 | 0 | 0 | 0 | 0 | 0 | 0 |
| AE leading to withdrawal of study treatment | 0 | 0 | 0 | 0 | 0 | 0 | 0 | 0 |
| AE resulting in death | 0 | 0 | 0 | 0 | 0 | 0 | 0 | 0 |
| ALL deaths | 0 | 0 | 0 | 0 | 0 | 0 | 0 | 0 |

Placebo: Matching Placebo is pooled across all treatment groups.

Group 1: ABBV-903 200 mg, IR Capsule.

Group 2: ABBV-903 400 mg, IR Capsule.

Group 3: ABBV-903 800 mg, IR Capsule.

Group 4: ABBV-903 800 mg, IR Tablet.

Group 5: ABBV-903 200 mg, IR Tablet.

**Table S3. Treatment-Emergent Adverse Events Reported by one or more Participants Administered Limnetrelvir or Placebo in Study 1 Part B**

|  | Placebo  (N=10)  N (%) | Group 1  (N=6)  N (%) | Group 2  (N=6)  N (%) | Group 3  (N=6)  N (%) | Group 4  (N=6)  N (%) | Group 5  (N=6)  N (%) | Total  (N=30)  N (%) | Overall  (N=40)  N (%) |
| --- | --- | --- | --- | --- | --- | --- | --- | --- |
| Participants with treatment emergent adverse event (AE) | 1(10.0) | 2(33.3) | 1(16.7) | 1(16.7) | 2(33.3) | 2(33.3) | 8(26.7) | 9(22.5) |
| AE with reasonable possibility of being related to study treatment | 0 | 1(16.7) | 0 | 1(16.7) | 2(33.3) | 1(16.7) | 5(16.7) | 5(12.5) |
| Severe AE | 0 | 0 | 0 | 0 | 0 | 0 | 0 | 0 |
| Serious AE | 0 | 0 | 0 | 0 | 0 | 0 | 0 | 0 |
| AE leading to withdrawal of study treatment | 0 | 0 | 0 | 0 | 0 | 0 | 0 | 0 |
| AE resulting in death | 0 | 0 | 0 | 0 | 0 | 0 | 0 | 0 |
| ALL deaths | 0 | 0 | 0 | 0 | 0 | 0 | 0 | 0 |

Placebo: Matching Placebo is pooled across all treatment groups.

Group 1: ABBV-903 200 mg QD, IR Capsule.

Group 2: ABBV-903 400 mg QD, IR Capsule.

Group 3: ABBV-903 200 mg QD, IR Tablets.

Group 4: ABBV-903 400 mg QD, IR Tablet.

Group 5: ABBV-903 600 mg QD, IR Tablet.

**Table S4. Treatment-Emergent Adverse Events Reported by one or more Participants Administered Limnetrelvir or Placebo in Study 2 Part A and 2 Part B**

|  | **Study 2A** | | | | | **Study 2B** | | |
| --- | --- | --- | --- | --- | --- | --- | --- | --- |
|  | Placebo  (N=4)  N (%) | Group 1  (N=6)  N (%) | Group 2  (N=6)  N (%) | Total  (N=12)  N (%) | Overall  (N=16)  N (%) | Placebo  (N=2)  N (%) | Group 1  (N=6)  N (%) | Total  (N=12)  N (%) |
| Participants with treatment emergent adverse event (AE) | 0 | 1(16.7) | 0 | 1(8.3) | 1(6.3) | 0 | 3(50.0) | 3 (37.5) |
| AE with reasonable possibility of being related to study treatment | 0 | 0 | 0 | 0 | 0 | 0 | 0 | 0 |
| Severe AE | 0 | 0 | 0 | 0 | 0 | 0 | 0 | 0 |
| Serious AE | 0 | 0 | 0 | 0 | 0 | 0 | 0 | 0 |
| AE leading to withdrawal of study treatment | 0 | 0 | 0 | 0 | 0 | 0 | 0 | 0 |
| AE resulting in death | 0 | 0 | 0 | 0 | 0 | 0 | 0 | 0 |
| ALL deaths | 0 | 0 | 0 | 0 | 0 | 0 | 0 | 0 |

Placebo: Matching Placebo is pooled across all treatment groups.

Study 2A, Group 1: ABBV-903 200 mg, IR Tablet.

Study 2A, Group 2: ABBV-903 400 mg, IR Tablet.

Study 2B, Group 1: ABBV-903 400 mg QD, IR Tablet.
